# Examining the Impact of the Golden Compass Clinical Care Program for Older People with HIV: A Qualitative Study

**DOI:** 10.1007/s10461-021-03509-0

**Published:** 2021-10-27

**Authors:** Judy Y. Tan, Meredith Greene, Cinthia Blat, Autumn Albers, Janet Grochowski, Jon Oskarsson, Mary Shiels, Priscilla Hsue, Diane Havlir, Monica Gandhi, Janet Myers

**Affiliations:** 1 Division of Prevention Science, Department of Medicine, University of California San Francisco, UCSF Box 0886, 550 16th Street, 3rd Floor, San Francisco, CA 94143 USA; 2 Division of Geriatrics, Department of Medicine, University of California San Francisco, San Francisco, CA USA; 3 Division of Maternal-Fetal Medicine, Department of Obstetrics, Gynecology and Reproductive Sciences, University of California San Francisco, San Francisco, CA USA; 4 Division of Cardiology, Department of Medicine, University of California San Francisco, San Francisco, CA USA; 5 Division of HIV, Infectious Diseases, and Global Medicine, University of California San Francisco, San Francisco, CA USA; 6 Facente Consulting, 5601 VAN FLEET AVE, 94804 Richmond, CA USA

**Keywords:** Older people with HIV, Aging and HIV, Clinical care models

## Abstract

The combined burden of geriatric conditions, comorbidities, and HIV requires a model of HIV care that offers a comprehensive clinical approach with people 50 years or older with HIV. *Golden Compass* is an outpatient, multidisciplinary HIV-geriatrics program with an onsite HIV geriatrician, cardiologist, pharmacist, and social worker, offering specialist referrals, care navigation, and classes on improving functional status and cognition. Participants (13 patients and 11 primary care providers) were recruited using a non-probability sampling method to participate in semi-structured interviews on the perceived impact of *Golden Compass* on care delivered to older people with HIV. Interviews were transcribed verbatim and framework analysis used to analyze the transcripts. The perceived impacts of *Golden Compass* by patients and providers were organized by the *Compass* points (Northern: Heart and Mind, Eastern: Bones and Strength, Southern: Navigation and Network, Western: Dental, Hearing, and Vision). Overall, patients valued the focus on functional health and whole-person care, leading to greater trust in the ability of providers. Providers gained new skills through the geriatrics, cardiology and/or pharmacist consultations. The HIV-geriatrics specialty approach of *Golden Compass* improved functional ability and quality of life for older adults with HIV. Few integrated care programs for older people with HIV have been evaluated. This study adds to the limited literature demonstrating high patient and provider satisfaction with a HIV-care model that incorporated principles of geriatric medicine emphasizing a comprehensive approach to sustaining functional ability and improving quality of life.

## Introduction

People with HIV are living longer. Worldwide, an estimated 7.5 million people with HIV are ages 50 years or older [1]. We use the age of 50 to characterize “older” for people with HIV due to the risk of age-related comorbidities and geriatric conditions at younger ages relative to the general population [2–6]. Managing comorbidities and geriatric conditions requires specialized treatments that in turn put older people with HIV at risk for other clinical complications, including drug-drug interactions [7–9]. Thus, the combined burden of geriatric conditions, comorbidities, and HIV requires a model of HIV care that offers a comprehensive clinical approach to complex clinical cases [10–13].

**Fig. 1:**
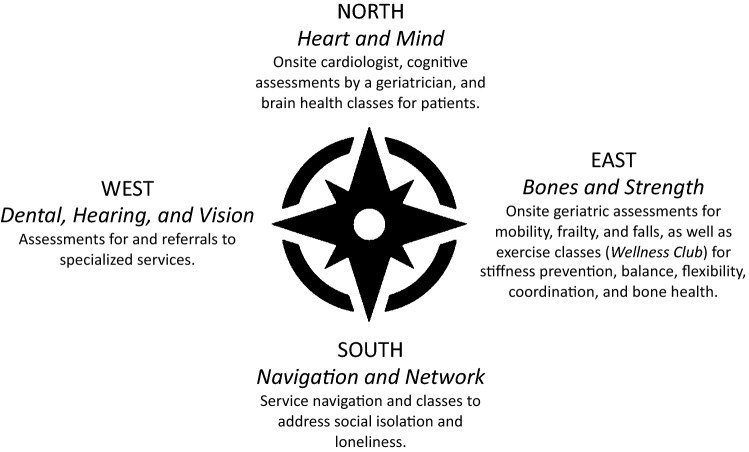
Four compass points of the Golden Compass Program, Ward 86, Zuckerberg San Francisco General Hospital

*Golden Compass* at Ward 86, a safety-net outpatient HIV clinic, was developed in response to the need for a broader care approach with older people with HIV (Fig. 1) [14, 15]. The *Golden Compass* multidisciplinary team includes a HIV geriatrician, a cardiologist, a pharmacist, and a social worker. Additional services include specialist referrals and navigation; classes on improving functional status and cognition [14, 15].

While research has characterized the health care needs of older people with HIV, much less is known regarding the impact of care programs designed and implemented to specifically address these needs. The perspectives and preferences of patients and care providers are often missing even as they should guide the evaluation of care models [16, 17]. The goal of this study was to explore the impact of *Golden Compass* from the perspectives of both patients and primary care providers at the Ward 86 HIV clinic using qualitative research methods.

## Methods

### Procedures

Participants (both patients and their primary care providers) were recruited for a one-time semi-structured interview at a metropolitan HIV clinic with 39 providers serving 2400 patients, most of whom are on public insurance or are uninsured. Interviews were conducted between October 2018 and May 2019 by authors JYT, CB, and JM. Non-probability sampling was used, resulting in a convenience sample of those who were easy to contact and engage. Patients were recruited via posted flyers and word of mouth; they were eligible for the study if they were living with HIV, 50 years of age or older, and had participated in at least one *Golden Compass* consultation or class. Individuals were screened by phone and, if eligible, were scheduled for an in-person or phone interview. Providers who referred at least one patient to the program were recruited via “Dear Provider” emails. Interviews were conducted in person or via phone or videoconference. Informed consent was obtained prior to the interview. Interviews were recorded for later transcription and each lasted 45–60 min. Participants received a $20 gift card. Study procedures were approved by the University’s Institutional Review Board.

Separate interview guides were developed for patients and providers. Interviews among patients explored experiences with *Golden Compass* and impacts on health. Among providers, interviews focused on the referral experience and their perspectives on the usefulness of the geriatrics and cardiology consultations, including observed impacts on patient health and on the provider’s care approach.

### Analysis Approach

Interviews were transcribed verbatim by a professional transcription service as they were completed. Transcripts were independently read by authors JYT, CB, JM, M. Greene as they became available, and interviews were conducted until thematic saturation was reached (i.e., no new information was gained from an additional interview) [18]. Themes were identified based on this preliminary reading of transcripts and were discussed and refined during regular team meetings. Data analysis was conducted separately by patients and providers via two approaches. First, analytic memos were developed independently by JYT, CB, JM who read and reread the same set of patient and provider transcripts and then met to discuss emergent themes and other observations [19, 20]. Next, JYT conducted framework analysis by developing data matrices based on themes by patient and provider and by which to organize the raw data, allowing comparisons of quotes across themes and participants [21]. These analyses were presented and discussed during regular team meetings.

## Findings

A total of 13 patients with HIV and 11 primary care providers were included in the study. Patients with HIV self-identified as the following: 11 (84.6%) as male, two (15.4%) as female, six (46.2%) as Black/African American, three (23.1%) as “Other”, three (23.1%) as White, two (15.4%) as Latinx, and one (7.7%) as Native Hawaiian/Other Pacific Islander. Demographics of primary care providers are not presented in order to protect the confidentiality of the small group of providers. Below, we first present findings on the overall experience of *Golden Compass* by patients and then providers. Next, we discuss respective patient and provider experiences (Table 1) organized by the *Compass* points (Fig. 1).

**Table 1:** Excerpts from patient participants (n = 13) and provider participants (n = 11), Golden compass program, San Francisco

Golden compass point	Theme	Excerpt
Participants who were patients with HIV		
NORTH: heart and mind	Engaging with other older people with HIV in the memory classes presented many patients the opportunity to practice skills learned from class	It was the memory class of how to remember people's names. I've always had a problem with that. So they taught us how to remember people's names by making up stories about each other’s names. < laughs > That was really fun. And it really did work. And now when I try to remember people's names I have to think of something funny. < laughs > My memory is getting a lot better
East: bones and strength	The geriatrician’s assessment led to improvements in ankle strength and balance	[The HIV geriatrician] found that I had a balance issue on my right side and she sent me to PT for it and so they taught me strengthening exercises…I never knew that and so if someone hadn't have said, "You've got some balance issues," and we're going to send you to PT to help with it [I’d never have done]
	Patients saw significant improvement in muscle and bone strength from participating in exercise classes (“*Wellness Club”)*	I wake up in the morning my ankles might hurt so bad I don't think I could walk on them. I was having problems just moving around and I’ve fallen a few times because my ankles had just given out on me. I’ve fallen on my knees a lot of times. So the very first day I got there they were doing this exercise… And ever since I've been doing it, my ankles feel so good
South: navigation and network	Classes not only improved individual outcomes such as memory and concentration (“*Brain Health*”) or physical strength (“*Wellness Club*”), but also provided opportunities to socialize with others who share similar experiences	This “*Wellness Club”* every Wednesday, we've all become friends with each other, we see each other around town, we see each other out in the street, we see each other in the cafeteria at different clinics for appointments and it's just another group of people that you know there's this commonality of us all getting together
	Beyond improving balance and strength, the benefits of physical strength and exercise classes (“*Wellness Club*”) included improvements in mental health symptoms and mood	I really like the Wellness Club. What I learned about exercise is that when I'm depressed, it actually helps my mental health. I get more upbeat—it just makes me want to stay healthier. It improves my mood. My body feels better, it just makes me forget about my depression, just getting out of the house and talking to people… My mood improves just by doing the hour of mobility without stopping. I never had an exercise group in the past. I like the music because we get our boogie on and shake—everybody dancing. I look around at everybody doing their moves that everybody be laughing, and then we hear something like that we like, and, you know, everybody, hey, we be giggling and just having fun. I like seeing people happy and dancing
	*Telling Your Story* classes addressed social isolation, helping patients find words for their own story connecting them to others, which help older people with HIV feel less isolated	I like the name, *Telling Your Story,* because I think that a lot of us are isolated. Our conversation is allowing me to say out loud for the first time many things that were in my head. I actually didn't know it until I put it together to tell you. And so that's sort of like learning what your own story is. And especially when you are disconnected from other people, knowing what you think your story is really important to just simple decision making and motivation. When people get together in a group, a lot of times they can—knots get untied
		For myself, it's a great benefit. That's why I attend regularly. I've made a connection with a lot of people, and I feel like they benefit from knowing me and I benefit from knowing them. The class is beneficial for me because I'm able to reflect on my life. I love hearing everybody's journey in life, and everybody's very receptive of everybody else's story
West: dental, hearing, vision	Navigation to services such as dental care underscores for patients that living longer with HIV means taking a whole-person approach to self-care	…My girlfriend we both got it in in 1999. She passed away in 2004. So who cares about your back and who cares about your eyes and your ears at that time. Everything else goes on the wayside… But now I’m healthier than I was even three years ago. I'm taking care of things. And my teeth I’m taking care of them. I've got to get this. There was a time that I would say why bother?
		The pharmacist, first off, told me that we’re going to go through my pill selection and see if I need everything. And then, like I said, we’re going to the eye doctor, ear doctor, all of those doctors to see if they're going to change anything, add anything, take away anything. We got that done
Participants who were primary care providers		
North: heart and mind	Cognitive assessments were helpful in addressing common issues such as mental “fogginess” due to side effects of medications to treat HIV-related symptoms	I think the cognitive assessment piece has been valuable. [The HIV geriatrician] will suggest maybe trying to decrease certain meds. Sleeping, pain with AIDS, or opiates that might be adding to the fogginess. And I think that's been really helpful to have a second voice, both in my head, and in my patient's head about how to address the concerns they have with memory and focus by thinking about the meds. Even if they've been on them for a long time, are things that are maybe having effect
East: bones and strength	The physical therapy and exercise classes recommended from the geriatric consultations were part of what improved strength and mobility for one patient. The following account from a provider corroborated the patient’s view	This kind of gait/stability assessment piece—I'm more conscious of it. How quickly can people get up out of chairs? Do they need to be using mobility assistant devices? I think I've definitely been keyed into that. And because of the assessments from the clinic. So that was the one guy. He was assuming his pain was all HIV/neuropathy. But after the visits with Golden Compass, [the HIV geriatrician] had a whole different take on it. Sent him to a podiatrist. He got these insoles and a whole different diagnosis of a tendonitis type thing
South: navigation and network	Collaboration between providers and pharmacists helps to ensure that patient medication regimens are as beneficial and streamlined as possible	When [the pharmacist and geriatrician] review the med list and say, “Look, hey, does this person really need the nortriptyline?” or “There’s these two meds in combination that may not be ideal” and suggest other things. It’s nice to have input on meds that potentially could be changed or are not necessary, and so I really appreciate that
West: dental, hearing, vision	Referrals and navigation to ancillary services provide crucial support for providers to address comorbidity and other issues commonly experienced by older patients with HIV	I mean, I think management of their comorbidities, right? It often then becomes not their HIV but all the other medical issues that they're having. Cognitive issues. And then all the sort of checklist of geriatrics, like gait, falls, vision, hearing, and then polypharmacy

### Overall Impact of Golden Compass

Patients felt that *Golden Compass* resulted in them having greater confidence in the quality of care received. Narratives centered on having greater confidence and, as a result, more engagement in their care as a result of participating in *Golden Compass*. Patients appreciated that their care was focused not around disease alone, but around functional health and a full spectrum of needs above and beyond HIV:

Dr. [HIV geriatrician], the Golden Compass…addresses more than my HIV. [The geriatrician] breaks it down with different doctors that you have to see. My [HIV] doctor does not address the cardiology and with the bone density and there is more than HIV with my health going on. So [the HIV geriatrician] addresses all the other problems I have going on, so it’s like more broad, more wider point of view.

Greater confidence in the quality of care received promoted more engagement in care among patients. One patient described how the experience of shared-decision making with the HIV geriatrician motivated him to become a better self-advocate in his overall care plan:

[Although] I’ve been receiving excellent care and things are well-managed, I’m not thinking about my health much at all. Dr. [HIV geriatrician] was talking to me about all the things that are set up to see if it could be improved. She empowered me to go to my doctor and advocate more strongly. I feel more empowered as a patient, and I feel like my medical care is more finely tuned. I have more confidence in the care that I’m getting...

Geriatric consultations also addressed self-management skills that patients found useful for managing comorbidities, as illustrated in the following passage:

When she asks you what’s wrong with you, she actually writes down a list... When I got home, I went over the list, and then I see what I got to do... She wrote down about doing the bone density and all the health issues I had going on, so it was easy for me to check on it because I went through my list and then I just had to call the numbers and make appointments. It was really helpful and the way she broke it down, it was really easy for me to… make my appointments. Otherwise I wouldn’t have done it myself.

Having a previously unaddressed health issue treated by *Golden Compass* was a new experience for many patients. One patient attributed the overall improvement in her functioning and quality of life to having a common age-related health issue (e.g., bladder control) addressed for the first time by the HIV geriatrician.

When Dr. [HIV geriatrician] asked the urination questions—nobody had ever done that before. She made sure that I understood that I had to train my bowels and train my urination... And everything she said worked. So now not only can I walk further, faster, I don’t have to use the restroom every hour.

For providers, consulting with the onsite geriatrician, cardiologist, and/or pharmacist led to new insights and skills for treating older patients with HIV, resulting in greater confidence in the management of older patients. Several emphasized that the geriatrics consultations enhanced treatment approaches with additional strategies and tools (e.g., cognitive screening, frailty assessmets) for addressing common issues (e.g., cognitive decline, falls).

I think [Golden Compass] is a really great resource because Dr. [HIV geriatrician]’s expertise is these older patients and they have some needs that we may not be addressing, [such as] looking at some medications that may be impacted by their decreased ability to metabolize… Am I missing something? Could I be thinking of something else? At times [HIV geriatrician] is familiar with resources in the community that might be helpful for a patient that I may not be aware of.

Providers also observed that the positive impacts of *Golden Compass* on patients’ health bolstered patients’ confidence in their care. A provider explained how improvements from the medication reconciliation from *Golden Compass* promoted care engagement by the patient:

I have one patient who is pretty frail and has a whole bunch of medical problems. Having *Golden Compass* say, “No, not that medicine, too risky, and please focus on these things and do this and come back in a month and check-in with me”…has been really good for his health actually, that I think he has a lot more confidence in his medical care and that he’s being listened to and seen and paid attention to and therefore has been a little more willing to change some of the ways he’s been doing his healthcare...

### Northern Point: Heart and Mind

#### Heart

The cardiology consultations increased patients’ awareness of cardiovascular health risks as a result of aging and living with HIV. In the following passage, a patient attributed his recovery from myocardial infarction to the care he received from the on-site cardiologist:

I’ve been seeing Dr. [Cardiologist] every two or three months. She takes a lot of worry out of what I have, [the myocardial infarction that] happened, and she explained to me that as long as I take care of my business, stay on it and don’t do anything negative about it, cigarettes or drinking or whatever, right? She’s been keeping me on the straight line in taking care of my heart and keep living.

For providers, the cardiology consultations were impactful in rounding out their skillset and care approaches, as well as in addressing comorbidities and polypharmacy:

I think patients have really enjoyed seeing Dr. [Cardiologist] and having that service on site... [The consultation] was really great: what medications to add, what workup to do. When I saw the patient after he had seen [the Cardiologist], he was able to articulate why she wanted to add this medicine, this medicine and do this test so he I think got a lot out of the visit other than just, “Oh, take these medications.”

#### Mind

Cognition and memory classes offered by *Golden Compass* were highlights for many patients. Patients noted improvements in memory and concentration as a result of participating in classes such as “*Brain Health*.” One patient learned specific skills not only for recall, but also for managing feelings of frustration around not remembering:

I’ll let a drop of the pin get me all upset when I can’t remember… I asked, why is it that I can go upstairs and think of something I need downstairs but by the time I get down there I have forgot. The teacher said that is called short-term memory. She said, “You know what you should do? Just stand there and just think. Eventually it will come to you.” I’m telling you I have learned so much in that one class...

For providers, the detailed advice given by the geriatrician and additional cognitive assessments were crucial in delineating specific next steps to address multimorbidity, as illustrated in the following:

As a medical provider, when you get all the comorbidities, how to address each of those but then how to bring those things all together in the aging person. There’s been a couple of very complex patients with comorbidities that I just kind of wasn’t sure where to go with and Dr. [HIV geriatrician] was able to kind of give some good direction on what kind of referrals might be needed. [F]or me big issues are cognition so her assessment, being able to spend more time and doing a more detailed assessment and then her recommendations on next steps is super helpful.

### Eastern Point: Bones and Strength

On-site geriatric assessments for mobility, frailty, and falls often uncovered underlying health issues. A patient related how a neurological exam initiated by the HIV geriatrician led in a diagnosis and subsequent treatment to slow the progression of an underlying condition:

[The HIV geriatrician] directed me to a consultation with a neurologist. Turns out, I have what they called a non-essential tremor in my right hand. And I’ve had that for years and, when that got worse and I fell, then they did these tests and came up with the diagnosis of early Parkinson’s. So I started taking medication for that.

Providers corroborated this view. A provider discussed below how specialty consultations were particularly critical to uncovering root causes of recurring falls, such as a slow heart rate:

I had not seen him for a while and he’d had an EKG after I’d seen him in clinic, and then had a fall and [she] noticed his [cardiac] rhythm was very slow and referred him for a pacemaker, which potentially... saved him from some illness. [Now] he’s stopped having falls, which was one of the reasons that brought him into medical care and we couldn’t figure out why at first...[H]e’s been stable, doing great, independent in the community. Certainly, he is very medically frail, so staying the same, minus falls, is a really good outcome for him, staying independent, living where he wants to live.

Patients described improvements in mobility, posture, agility, daily functional status, and pain as a result of participation in the *Wellness Club* exercise classes:

I have noticed changes in my physical wellbeing. [B]efore, I wasn’t really as active. Now, when I do that, my body’s not as stiff, it’s not as sore, I’m more limber, and that helps me in my life. If I didn’t have that, then I would probably be in a worse situation than I am.

### Southern Point: Navigation and Network

#### Navigation

Patients described the benefits of accessing co-located services at a familiar location through the Program:

There is an advantage…because it’s communication between the [primary] doctor, the pharmacist, the HIV-geriatrician, and then myself. So, it’s kind of like teamwork, and that teamwork is all there to help us. They’re centrally located, I think that’s a great benefit.

Patients appreciated that the pharmacist reviews their medications and obtains input from the patient’s care team in modifying their medication regimen, which reduced pill burden. Collaboration between providers and the pharmacist helps to ensure that patient medication regimens are streamlined.

#### Network

While classes did not focus specifically on addressing social isolation, they naturally provided a forum for building new relationships and social connections. Classes not only improved individual outcomes such as memory and concentration (“*Brain Health*”) or physical strength (“*Wellness Club*”), but also provided opportunities to socialize with others who share similar experiences, as in the narratve story-telling class called *Telling Your Story*. Engaging in personal storytelling with other older people with HIV helped one patient feel less stigmatized and isolated as illustrated by the statement below:

As we get older, we lose a lot of our friends, and we’re just left alone with our minds thinking about the past, and that becomes a big issue for people like me. The class has helped me a lot because I get to meet other people that are just like me, and that, I think, is very healthy—to connect to other individuals that are going through the same things.

### Western Point: Dental, Hearing, Vision

The Western point of *Dental, Hearing, Vision* refers to care navigation and referrals made to specialists outside of our clinic that are essential to the health of older individuals. Consultations led to timely access to a wide range of services and referrals:

[The HIV geriatrician] was a type of doctor that teaches you how to get to a specialist. She hooked me up with the eye doctor, the ear doctor, a back doctor and a pharmacist all at the same time. It’s like within a month I had seen them all. And where previously that would take nine months to see all four doctors; all four of the same doctors at once that would’ve taken nine months.

Providers appreciated the referrals and navigation to specialty services that help guide them in accessing specific resources and services, which can be challenging:

You know, dental, I’m not sure how much they do and as primary care, this is one of the hugest things that’s an obstacle is that you have to print all this stuff, you have to have it faxed to the dental school, you have to call the patient and the systems aren’t real good.

## Discussion

The broad and different care approaches of *Golden Compass* at Ward 86 were credited by both patients and providers for improvements across health domains, including daily functioning and quality of life. Patients perceived improvements in memory, social isolation, falls, physical pain, walk/gait, navigation, polypharmacy, and skills for co-managing other chronic conditions. Geriatric, cardiology, and pharmacy consultations improved patient-reported outcomes and raised provider knowledge and confidence in caring for older patients. Navigation to dental, hearing, and vision services off site enhanced the whole-person care approach. Patients’ improved functional status and quality of life also contributed to greater trust in their care overall and in their willingness to participate in shared decision making.

Few integrated care programs for older people with HIV have been evaluated. The findings in this and in previous assessments of *Golden Compass* [15] are consistent with the limited literature demonstrating high patient and provider satisfaction with care models that incorporated geriatric medicine in HIV care [22, 23]. Principles of geriatric medicine emphasize a comprehensive problem-solving approach to sustaining functional ability and improving quality of life [22, 23]. Our findings illustrate that the *Golden Compass* multidisciplinary team embedded within an HIV clinic contributed to self-reported improved knowledge and confidence among providers in caring for patients, which aligns with other models of HIV care for older adults involving both a general practitioner and specialists [22].

Participant narratives commonly related to two aspects of *Golden Compass*—co-location of services and a positive patient-provider relationship—that facilitated their care experience. Co-location of services improved timely access to a wide range of services and supported coordination of holistic care across providers, whereas positive patient-provider relationships fostered trust and empowered patients to advocate for their care. These findings are consistent with prior research demonstrating that patients with HIV value care coordination, inter-service communication, ease of access to services, a good health care professional-patient relationship, and involvement in decisions about care [16, 17]. Research has also emphasized the importance of one-stop, co-located services with diverse teams of clinical and nonclinical providers to provide successful interdisciplinary HIV care [24].

The co-location of services and enhanced patient-provider relationships described by participants may help explain their perceived improvements in health outcomes. A systematic review of 36 studies in which HIV services were co-located with other services showed some evidence for the association between co-location and improved HIV outcomes [25]. Among 10 studies in which non-HIV specific services were paired with HIV primary care, eight (80%) of studies reported positive associations across an HIV-related outcome and two (20%) reported no effect [25]. With respect to patient-provider relationships, high-quality relationships, where a provider knows a patient “as a person,” are associated with higher odds of HIV treatment adherence and viral suppression [26]. Explanatory modeling suggests that patient-provider interactions directly and positively impact patient satisfaction, medication adherence, and quality of life [27]. Furthermore, conceptual models highlighted the important of dyadic trust between patients and providers in facilitating adherence to non-HIV specific medications among people with HIV [28]. While research on patient-provider relationships among older adults with HIV is lacking, studies of older adults enrolled in Medicare have demonstrated that higher quality patient-provider relationships are associated with patients being more active in healthcare decisions [29]. Higher engagement in health care decision making—engagement that is enhanced by *Golden Compass*—has been associated with better quality of life indicators, including mental, physical, cognitive, and behavioral outcomes, among people with HIV [30].

### Limitations

A convenience sample was recruited and may not be representative of the entire patient population 50 years or older at Ward 86. However, the study was designed to offer insights to inform future research directions, an inductive research approach for which a purposive sampling method was appropriate.

### Conclusions

The population of people ages 50 years or older with HIV is growing and requires unique models of care to address specific health challenges currently further exacerbated by the COVID-19 pandemic. While such models are emerging, little is known regarding their impact on patient outcomes and the way that primary care providers manage older patients’ care. This study explored the perspectives of patients and their providers regarding their experiences with *Golden Compass*, a multidisciplinary program serving people with HIV 50 years or older at a safety-net HIV clinic. Findings highlighted the positive impacts of co-location and that *Golden Compass* improved patient trust, which in turn enhanced patient-provider relationships. Future research should evaluate this and other HIV and aging programs for improving patient outcomes over time.

## Data Availability

Data associated with this manuscript will not be deposited.

## Code Availability

There were no software applications or custom codes associated with this study/manuscript.
